# Multiple Pathways Involved in Palmitic Acid-Induced Toxicity: A System Biology Approach

**DOI:** 10.3389/fnins.2019.01410

**Published:** 2020-01-31

**Authors:** Daniel Osorio, Andrés Pinzón, Cynthia Martín-Jiménez, George E. Barreto, Janneth González

**Affiliations:** ^1^Department of Veterinary Integrative Biosciences, Texas A&M University, College Station, TX, United States; ^2^Laboratorio de Bioinformática y Biología de Sistemas, Universidad Nacional de Colombia, Bogotá, Colombia; ^3^Departamento de Nutrición y Bioquímica, Facultad de Ciencias, Pontificia Universidad Javeriana, Bogotá, Colombia; ^4^Department of Biological Sciences, University of Limerick, Limerick, Ireland; ^5^Health Research Institute, University of Limerick, Limerick, Ireland

**Keywords:** tibolone, astrocytes, inflammatory response, flux balance analysis, palmitic acid, genome-scale reconstruction, systems biology

## Abstract

Inflammation is a complex biological response to injuries, metabolic disorders or infections. In the brain, astrocytes play an important role in the inflammatory processes during neurodegenerative diseases. Recent studies have shown that the increase of free saturated fatty acids such as palmitic acid produces a metabolic inflammatory response in astrocytes generally associated with damaging mechanisms such as oxidative stress, endoplasmic reticulum stress, and autophagic defects. In this aspect, the synthetic neurosteroid tibolone has shown to exert protective functions against inflammation in neuronal experimental models without the tumorigenic effects exerted by sexual hormones such as estradiol and progesterone. However, there is little information regarding the specific mechanisms of tibolone in astrocytes during inflammatory insults. In the present study, we performed a genome-scale metabolic reconstruction of astrocytes that was used to study astrocytic response during an inflammatory insult by palmitate through Flux Balance Analysis methods and data mining. In this aspect, we assessed the metabolic fluxes of human astrocytes under three different scenarios: healthy (normal conditions), induced inflammation by palmitate, and tibolone treatment under palmitate inflammation. Our results suggest that tibolone reduces the L-glutamate-mediated neurotoxicity in astrocytes through the modulation of several metabolic pathways involved in glutamate uptake. We also identified a set of reactions associated with the protective effects of tibolone, including the upregulation of taurine metabolism, gluconeogenesis, cPPAR and the modulation of calcium signaling pathways. In conclusion, the different scenarios studied in our model allowed us to identify several metabolic fluxes perturbed under an inflammatory response and the protective mechanisms exerted by tibolone.

## Introduction

Astrocytes are the most abundant cells in the human brain. In the last years, it has been shown that they are of paramount importance for different essential functions in central nervous system (CNS). For instance, the homeostatic regulation of the central nervous system (Takuma et al., 2004), tissue repair, modulation of synaptic activity through the release of gliotransmitters and glycogen storage (Lange et al., 2012). Additionally, astrocytes protect neurons against the glutamate-induced excitotoxicity through the astrocyte-specific sodium-dependent glutamate transporters such as GLT-1 and GLAST (Bélanger and Magistretti, 2009). On the other hand, astrocytes are important modulators of inflammation (Sofroniew, 2014). Their main response to inflammation happens through the activation of the complex process of reactive gliosis, which is an important process for CNS during injuries and diseases (Dowell et al., 2009; Barreto et al., 2011; Sofroniew, 2014). For these reasons, a great number of studies have shown that the dysregulation of astrocytic functions is highly correlated with the development of neurodegenerative processes, (Takuma et al., 2004; Kumar Jha et al., 2016).

Different works have shown that astrocytes are key mediators in the brain lipid homeostasis and B-oxidation of fatty acid (Panov et al., 2014). Interestingly, saturated free fatty acids, including stearic acid, lauric acid, and palmitic acid, are closely associated with neurodegenerative processes such as traumatic brain injury (TmBI), dementia, stroke, epilepsy, spinal cord injury, Parkinson’s disease (PD) reactive gliosis, neuroinflammation, and Alzheimer’s disease (AD) (Bruce-Keller et al., 2001, 2009; White et al., 2009; Gupta et al., 2012; Little et al., 2012; González-Giraldo et al., 2019). Additionally, both palmitic acid and stearic acid were shown to increase the secretion of Aβ amyloid peptide in an AD cellular model (Amtul et al., 2011). Recent studies in human populations also point an inverse correlation between clinical obesity and neuroinflammation (Barnard et al., 2014; Reichelt et al., 2017; Melo et al., 2019), suggesting that long-term consumption of high fat diets is associated with pathological mechanisms in the brain (Melo et al., 2019). Moreover, the increase of saturated free fatty acids during metabolic inflammation activates IKKβ kinase and its downstream effector NF-κβ, which in turn impairs leptin and insulin hormonal signaling and triggers the production and release of reactive oxygen species (ROS) and pro-inflammatory cytokines like TNF-α and IL-6 from glial cells (Purkayastha and Cai, 2013).

In this aspect, tibolone is a synthetic steroid (Kloosterboer, 2001), with estrogenic, progestogenic, and weak androgenic actions (González-Giraldo et al., 2019). It has been used for the treatment of climacteric symptoms and osteoporosis in post-menopausal women (Rymer et al., 1994; Rymer, 1998) and has also shown beneficial antidepressant effects in menopausal women (Kulkarni et al., 2015). It has been shown that tibolone exerts its neuroprotective effects through the activation of the Akt/GSK3ß signaling pathway which in turns causes the reduction of Tau phosphorylation in the hippocampus and cerebellum of ovariectomized rats, the increase in antioxidant activity in primary neuronal cultures and the increase in the expression of the antiapoptotic protein Bcl-2 (Genazzani et al., 2006; Belenichev et al., 2012; Pinto-Almazán et al, 2012; Avila-Rodriguez et al., 2014). Nevertheless, there is little information regarding the effects of tibolone in astrocytes or the metabolic pathways related with its neuroprotective mechanisms (Avila-Rodriguez et al., 2014; González-Giraldo et al., 2019).

In that sense, genome-scale metabolic reconstructions are a compilation of all the stoichiometric reactions and pathways that can describe the entire cellular metabolism of an organism (Vodovotz et al., 2008; Thiele et al., 2013). In recent years, they have become an indispensable tool for the understanding of complex biological phenomena, including neurodegenerative diseases and inflammation processes (Cakir et al., 2007; Swainston et al., 2013; Sertbaş et al., 2014; Martín-Jiménez et al., 2017). Moreover, genomic-scale reconstructions are builder from a system biology approach that allows the integration of several sources of information, such as biological data bases, high-throughput omic data, and experimental evidence, in order to improve the development of novel pharmacological treatments (Najafi et al., 2014).

Having in account the importance of astrocytes for brain inflammation, and the promising effects of tibolone for astrocytic and neuronal protection (Crespo-Castrillo et al., 2018), we developed a genomic-scale metabolic model of astrocytes, with the purpose of enlighten the metabolic pathways modulated by tibolone during an inflammatory response caused by the increased uptake of palmitate. We focused or attention, in the identification of metabolic changes related with the modulation of cytokines, the release of gliotransmitters and the neuroprotective effects mediated by tibolone in an inflammatory scenario (Wojtal et al., 2006). Our results suggest that tibolone exerts its neuroprotective effects through the reduction of neurotoxicity mediated by L-glutamate in astrocytes. We also found a tibolone-associated increase in the biomass growth rate that is consistent with previous reports concerning the side effects of neurosteroids in other human cell types.

## Materials and Methods

### Tissue Specific Model Construction

The tissue specific model construction process started with the identification of all enzyme-coding genes expressed in healthy human astrocytes indexed in the GEO database (Swainston et al., 2013) as GSE73721. Gene identifier conversion from GeneCards (Rebhan et al., 1997) to ENTREZ (Maglott et al., 2011) was performed through “UniProt.ws” R Package. Reactions associated with the identified genes were mapped from the Human Genome-Scale Metabolic Reconstruction RECON 2.04 (Thiele et al., 2013) as downloaded from the VMH Lab (Swainston et al., 2016)^1^ and further enriched with metabolic information obtained from KEGG (Kanehisa and Goto, 2000). Additionally, we developed the R package “g2f” available in CRAN (Hornik, 2012; Botero et al., 2016) to identify and fill the gaps using all the reactions with an uncorrelated gene expression included in RECON 2.04, as well as to select and remove all blocked reactions from our reconstruction.

All the reactions involved in the conversion of extracellular glutamate, glycine, cysteine and glucose to extracellular glutamine, glycine, serine-D, reduced glutathione, lactate, and ATP were added, as they are essential for the normal astrocytic metabolism (Barreto et al., 2011; Souza et al., 2019). Exchange reactions were limited to components of the Dulbecco’s Modified Eagle Medium (DMEM) as an input, and the gliotransmitters, glutamine, D-serine, ATP and glutamate, reduced glutathione, lactate, glucose, nitric oxide, prostaglandins and leukotrienes as output, in accordance with previous experimental studies from our group (Avila-Rodriguez et al., 2016; Cabezas et al., 2018; González-Giraldo et al., 2019). Finally, we developed the R Package “MinVal” to validate the syntax of the model, the mass-charge and the creation of SBML files (Osorio et al., 2017).

Reaction limits (upper and lower bounds) were constrained proportionally to the mean gene expression reported for genes included in Gene-Protein-Reaction (GPR) (Thiele and Palsson, 2010) associated to each reaction in samples of male and female human patients from 47 to 63 years old, using the “exp2flux” R package available in CRAN (Hornik, 2012; Osorio et al., 2017). All Flux Balance Analysis (FBA) were performed using the “sybil” (Gelius-Dietrich et al., 2013) R Package running under R 3.3.1 (Gelius-Dietrich et al., 2013; R Development Core Team, 2016).

### Flux Balance Analysis

Flux balance analysis is a linear optimization method for simulating the metabolic reactions of a cell or an organism that allows the identification of the set of reactions involved in the production of a biological response within the metabolic model (Orth et al., 2010). The metabolic reactions are represented internally as a stoichiometric matrix (S), of size m×n, where m represents the metabolites and n the reactions. The entries in the matrix are the stoichiometric coefficients of the metabolites that take part in a reaction. The flux through all of the reactions in a network is represented by the vector v, which has a length of n. The concentrations of all metabolites are represented by the vector x, with length m.

The systems of mass balance equations at steady state are defined by:

$$\frac{d_{x}}{d_{t}}=0\,\mathrm{or}\,S\,\times \,v=0.\mathrm{FBA}$$

This expression seeks to maximize or minimize an objective function, which can be any linear combination of fluxes to obtain a flux for each reaction, indicating how much each reaction contributes to the objective function (Orth et al., 2010). The FBA for the studied scenarios was resolved using GLPK 4.60^2^, setting the generic human biomass reaction included in RECON 2.04 as default, and each one of the reactions described in Table 1 as objective functions. Models for each scenario were analyzed by comparing their specific fluxes, metabolite’s production rate and a sensitivity analysis.

**TABLE 1:** Set of objective functions used to evaluate the protective effects of tibolone in the inflammatory scenario.

**ID**	**Formula reaction**	**Description**
Glu2Gln	1 glu_L[e] → 1 gln_L[e]	Glutamate – Glutamine cycle
Gly2SerD	1 gly[e] → 1 ser_D[e]	Glycine to D-serine conversion
Glc2Lac	1 glc_D[e] → 2 lac_L[e]	Lactate production from glucose
Glc2ATP	1 glc_D[e] → 36 atp[e]	ATP production from glucose
Cys2GTHRD	1 cys_L[e] + 1 glu_L[c] + 1 gly[c] → 1 gthrd[e]	Catch of cysteine to produce reduced glutathione

### Identifying Flux Changes Between Scenarios

The measurement of flux change for each reaction between metabolic scenarios is a task generally carried out manually and oriented directly toward the research objective. However, at system level this process can become laborious. The *flux Differences function* calculates the fold change for each common reaction between metabolic scenarios.

*Fold change* is a measure that describes how much a quantity changes going from an initial to a final value. The implemented algorithm in the *flux Differences function* is described in Eq. 1:

$$\begin{array}{c} f\,o\,l\,d\,C\,h\,a\,n\,g\,e:\mathbb{R}\times \mathbb{R}\to \mathbb{R} \end{array}$$

(1) $$\begin{array}{c} \left(rFluxModel1,rFluxModel2\right) \end{array}\mapsto \begin{array}{l} \{\begin{array}{ll} rFluxModel2, & rFluxModel\text{1=0};\\ \frac{rFluxModel1-rFluxModel2}{|rFluxModel1|}, & \text{Othercases} \end{array} \end{array}$$

Here, the function takes as argument two valid models for the “sybil” R package and a customizable threshold value to filter functions to be reported.

In this aspect, we chose an arbitrary threshold value greater or equal to 2-fold times for reactions with an absolute change between the unconstrained and constrained metabolic scenarios, as reported in previous models (Hausen et al., 2015; Banos et al., 2017).

### Metabolic Scenarios

To test the protective effects of tibolone during metabolic inflammation in astrocytes we defined three different metabolic scenarios: (1) A “healthy” scenario, where the rate of palmitate uptake was set freely by the optimizer. This scenario emulates the normal conditions of astrocytes metabolism (Supplementary Data Sheet 1), without any inflammatory response (Seeger et al., 2016). (2) An “induced inflammation by palmitate” scenario, where the uptake rate of palmitate was forced to be stable in the mean of the half maximal inhibitory concentration (IC_50_) value for all the objective functions included in Table 1. In this aspect, IC_50_ values were calculated through a robustness analysis performed using uptake of palmitate (‘EXhdca(e)’ in RECON 2.04) as control and 1000 points in the range from 0 to 1 mMgDW–1h–1 for each objective function. Uptake values where each objective function reached IC_50_ was selected and subsequently averaged. Moreover, the modeled inhibitory effects are in congruence with the reported damaging effects of palmitate in astrocytes (Gupta et al., 2012; González-Giraldo et al., 2019). Finally, a “Tibolone treatment under inflammation” scenario was defined as an “inflammatory scenario” which included 279 additional reactions associated with the metabolic effects exerted by estradiol and derivates compounds obtained in KEGG (Kanehisa et al., 2014), and ten specific reactions associated with tibolone metabolism (Kloosterboer, 2004) not included in RECON 2.04, which are described in Table 2.

**TABLE 2:** Set of reactions added to recreate the “Tibolone treatment” scenario for the astrocytic model.

**ID**	**Formula reaction**	**Description**
T1	Tibolone[e] ↔	Tibolone exchange reaction
T2	Tibolone[e] ↔ a3OHtibolone[e]	3*a*hidroxytibolone interconvertion
T3	Tibolone[e] ↔ b3OHtibolone[e]	3*b*hidroxytibolone interconvertion
T4	Tibolone[e] → d4tibolone[e]	D4tibolone isomer formation
T5	b3OHtibolone[e] → d4tibolone[e]	D4tibolone isomer formation from 3*b*-hidroxytibolone
T6	a3OHtibolone[e] → estradiol[c]	Estradiol receptor agonist action mechanism of 3*a*-hidroxytibolone
T7	b3OHtibolone[e] → estradiol[c]	Estradiol receptor agonist action mechanism of 3*b*-hidroxytibolone
T8	d4tibolone[e] → prgstrn[c] + tststerone[c]	Progesterone and androgen receptor activation by tibolone D^4^ isomer
T9	a3OHtibolone[e] ↔ a3SOtibolone[e]	3*a*hidroxytibolone interconvertion to sulfated inactive compounds
T10	a3SOtibolone[e] →	Tibolone inactive form in blood

*Tibolone reactions affecting the astrocytic metabolism were based on Kloosterboer (2004).*

### Metabolic Changes

Flux differences for each reaction between optimized scenarios were measured using the fold change as described in the following equation:

(2) $$f\,o\,l\,d\,C\,h\,a\,n\,g\,e=\frac{v\,a\,l\,u\,e\,M\,o\,d\,e\,l\,2-v\,a\,l\,u\,e\,M\,o\,d\,e\,l\,1}{\left|v\,a\,l\,u\,e\,M\,o\,d\,e\,l\,1\right|}$$

### Mechanisms of Associated Enzymes With Pro-inflammatory, Anti-inflammatory and Tibolone Effects

Enzymes involved in pro-inflammatory and anti-inflammatory responses during palmitic acid damage and upon tibolone treatment were identified through a sensitivity analysis as follows: Pro-inflammatory enzymes are those that catalyze reactions that allow the increase of the objective function value when knocked out. Anti-inflammatory enzymes are those associated with reactions that have a fold-change greater or equal to 2, and that once being knocked out reduces the objective function value. Tibolone associated enzymes are those that catalyze reactions that produce a total inhibition of the metabolic effects of tibolone when knocked out.

## Results and Discussion

### Astrocytic Metabolic Model

**FIGURE 1:**
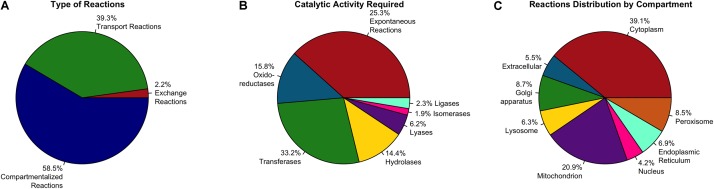
Distribution of enzymes included in the astrocyte metabolic model. Classification is based in **(A)** Type of reaction, **(B)** Catalytic activity and **(C)** Associated compartment of the enzyme (subcellular location).

We reconstructed an FBA based astrocytic tissue-specific model composed by 1262 unique genes, 1956 metabolites and 2747 biochemical reactions of which 1607 were intracellular reactions, 60 were exchange reactions, and 1080 were transport reactions (Figure 1A). Reactions were classified based on their enzymatic activity according to their EC (Enzyme Commission) numbers (Figure 1B), and sub-cellular localization (Figure 1C).

Based on the enzymatic classification (Figure 1B), 33.2% of total reactions are catalyzed by a transferase enzyme, 15.8% by an oxidoreductase, 14.4% by a hydrolase, 6.2% by a lyase, 2.3% by a ligase, 1.9% by an isomerase and 25.3% of them are spontaneous reactions without an associated enzyme or gene associated. Regarding subcellular localization, cytosolic and mitochondrial reactions contributed to 60% of the total reactions in the model. The remaining 40% of reactions are distributed in six subcellular compartments as follows: 8.7% in Golgi apparatus, 8.5% in peroxisome, 6.9% in endoplasmic reticulum, 6.3% in lysosome, 4.2% in nucleus, and finally 5.5% are transport reactions from the extracellular space (Figure 1C).

**FIGURE 2:**
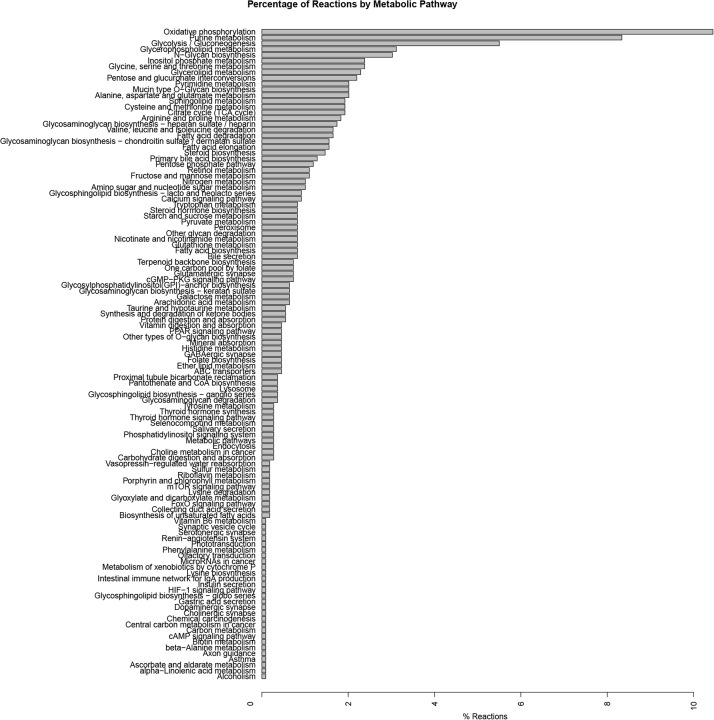
Summary of pathways associated with biochemical reactions included in the genome-scale metabolic model for astrocytes. Pathway association was assigned based in the metabolic categories used in the KEGG database.

The reactions included in the model are associated with 113 metabolic pathways reported in the KEGG database (Kanehisa and Goto, 2000). Almost 50% of reactions are associated to 10 main metabolic pathways of paramount importance for astrocytic metabolism and neuronal support such as oxidative phosphorylation, purine metabolism, glycolysis and gluconeogenesis, and pentose and glucuronate interconvensions. The distribution of reactions in metabolic pathways is shown in Figure 2. These results are similar to those previously reported astrocytic models (Fitch and Silver, 1997; Ciccarelli et al., 2001; Çakir et al., 2007; Giaume et al., 2010; Sertbaş et al., 2014; Martín-Jiménez et al., 2017; Sá et al., 2017). For example, Martín-Jiménez et al. (2017) developed a genome-scale metabolic reconstruction of human astrocyte, comprising of 5.659 reactions (237 exchange reactions and 1.948 transport reactions), 3.765 genes, 862 enzymes, 5.007 metabolites. Regarding the subcellular distribution of reactions, cytosolic and mitochondrial reaction accounted for 59% of the total reactions, while 23% belonged to peroxisome, lysosome, endoplasmic reticulum, golgi apparatus and nucleus. Finally, transport reactions represented 18% of the total reactions, making them highly similar to our model.

### Healthy Scenario (Basal Conditions)

**FIGURE 3:**
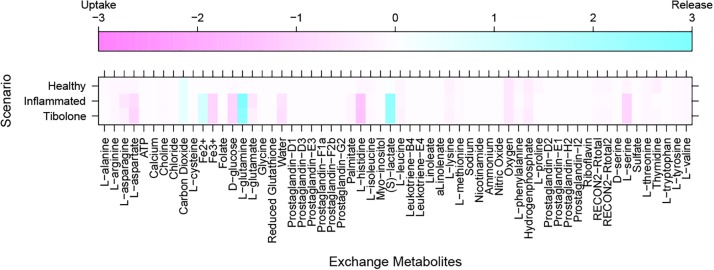
Exchange rate of metabolites between the different metabolic scenarios using the generic biomass reaction included in RECON 2.04, as the objective function.

**FIGURE 4:**
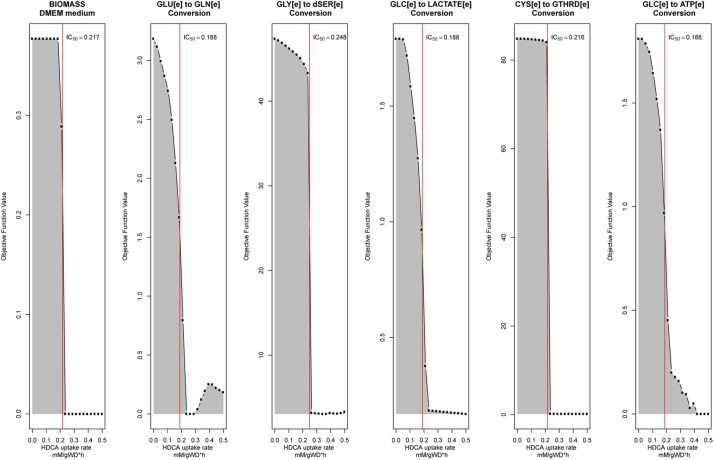
Robustness analysis to calculate palmitate-induced IC_50_ value for each objective function described in Table 1. The red line represents the calculated IC_50_ value.

Our metabolic simulation predicts a slow growth rate for astrocytes (0.37 mMgWD*-*1h*-*1) under normal conditions using DMEM medium as metabolic supply^3^. This result is in agreement with the study of Das et al. (2010) which reported that Human Normal Astrocyte cells (HNA) are able to grow in DMEM culture medium supplemented with 2% FBS (Das et al., 2010). Moreover, astrocytes activated 52% of model reactions (Figure 3) and preferentially use a glucose-based metabolism. Our model also shows that glucose is catabolized and constitutively released by astrocytes as lactate without any stimuli similar (Le Foll and Levin, 2016), in agreement with Cakir et al. (2007) and Bhowmick et al. (2015) whom reported a lactate release rate of 8.9% from the glucose flux in resting conditions (Çakir et al., 2007; Bhowmick et al., 2015). As previously stated, astrocytes in physiological conditions release large amounts of lactate to the extracellular space (Mangia et al., 2009), which can be used by neurons to supply their energetic requirements (Kumar Jha et al., 2016). Moreover, our simulations show that ATP and glutamate are synthesized and released by astrocytes only under the metabolic alterations present in the inflammatory and tibolone treatment scenarios which were analyzed through the evaluated objective functions of our model (Table 1). Metabolite release rate and biomass growth were used as references for the comparative changes between the three metabolic scenarios (Figures 3, 4).

### Inflammatory Scenario

In this scenario, we simulated an inflammatory microenvironment by increasing the cellular concentration of palmitic acid in astrocytes (Liu et al., 2013; González-Giraldo et al., 2019). The calculated IC_50_ for palmitic acid (Table 1) was 0.208 ± 0.024 mMgDW^–1^h^–1^, similar to the study by Liu et al. (2013) which used a concentration of 0.2 mM of palmitic acid to induce astrogliosis in primary rat cortical astrocytes (Liu et al., 2013). Upon palmitic acid, astrocytes increased the uptake of L-asparagine, L-aspartate, iron, D-glucose, L-glutamate, histidine and L-serine and the release of L-glutamine and lactate (Figure 3). This response is usual in reactive astrocytes under neuroinflammation, which leads to homeostatic disturbances, including an increased uptake in iron in CNS cells (Kumar Jha et al., 2016). Iron accumulation is present in several neurodegenerative diseases such as AD, and PD, promoting microglial pro-inflammatory activity, altering mitochondrial function, and inducing ROS production (Williams et al., 2012). An increase in histidine uptake was previously reported as a biomarker for metabolic inflammation during obesity (Niu et al., 2012). In this aspect, histidine acts as a free-radical scavenger that might reduce IL-6, TNF-α, and CRP levels, and inhibit the secretion of H_2_O^–^_2_ and TNF-α induced by IL-8 (Lee et al., 2005; Son et al., 2005). Aspartate, present in the brain as *N*-Acetyl-L-aspartate (NAA), is synthesized and stored in neurons but is hydrolyzed in glial cells (Baslow, 2003). NAA act as an anti-proliferative, antiangiogenic, and anti-inflammatory molecule by inducing the decrease of prostaglandin E2 (PGE2) in astrocytes (Rael et al., 2004). L-Asparagine, in turn, acts as a regulator of ammonia toxicity through the increase of Na^+^ intracellular concentration when is co-transported within astrocytes (Chaudhry et al., 1999). Moreover, asparagine induces a Ca^2+^ response comparable to GABA-induced Ca^2+^ transients in a dose-dependent manner (Doengi et al., 2009).

L-serine and L-asparagine uptake increase may be related to a cell survival process that switches cellular metabolism to be highly dependent of non-essential amino acids available in extracellular space such as glutamine, serine, glycine, arginine, and asparagine (Green et al., 2014). Moreover, under the inflammatory scenario, our astrocyte model released a limited amount of prostaglandin D2 (<1e-6 mMgDW-1h-1), a mediator of inflammation. It has been shown that reactive astrocytes express the DP_1_ receptor of prostaglandin D2, and that the inhibition of this receptor resulted in reactive gliosis suppression in mice (Mohri et al., 2006).

**FIGURE 5:**
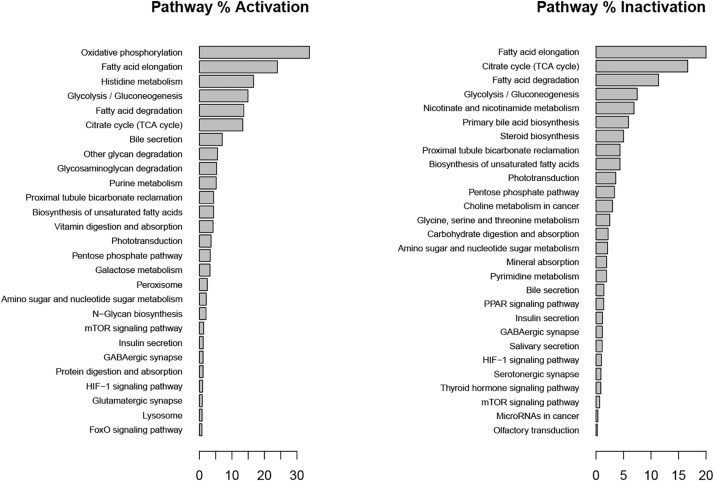
Metabolic pathways affected during metabolic inflammation by palmitate. Percentage of activation and inactivation was calculated compared with genes associated with each pathway in the KEGG database.

**FIGURE 6:**
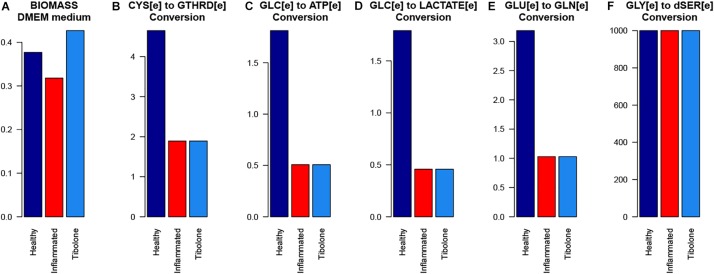
The response of the main astrocytes metabolic capabilities to different modeled scenarios. **(A)** Biomass with DMEm medium, **(B)** Cystenine to reduced glutathione conversion, **(C)** Glucose to ATP conversion, **(D)** Glucose to lactate conversion, **(E)** Glutamate to Glutamine conversion, and **(F)** Glycine to D-serine conversion.

In the inflammatory scenario, astrocytes modified the flux rate of 586 reactions when compared with the unstimulated scenario. Main metabolic changes are present in oxidative phosphorylation, histidine metabolism, and fatty acid degradation pathways, as well as the inactivation of TCA and glycolysis pathways (Figure 5). Inflammation affects all metabolic objective functions evaluated (Table 1) except for the release of D-serine. In this aspect, it was observed that there was a decrease of 15.6% in the growth rate of astrocytes compared with the normal scenario, a decrease of 59.3% in the intake of cysteine related to reduced glutathione production, a decrease of 72% in glucose degradation to ATP 72%, and to lactate in 74.4%. Finally, the conversion of extracellular glutamate to glutamine was reduced by 67.7% (Figure 6).

Based on the sensibility analysis, we identified two pro-inflammatory candidate reactions that were knocked out (Table 3). These reactions are associated with the formimidoyl-transferase cyclodeaminase (FTCD) and mitochondrial water transport, which has been associated with aquaporin-9 (Potokar et al., 2016). Following the inhibition of these reactions, it was observed an increase of the objective function above the maximum value set for the inflammatory scenario (11.45 and 5.14%, respectively). In this aspect, it has been shown that FTCD is overexpressed in high-fat diets (Fernando et al., 2013) and contributes with one-carbon units from histidine degradation to the folate pool (Väremo et al., 2015) and glutamate synthesis. Moreover, this enzyme has been associated with working memory performance in young adults (Greenwood et al., 2018). On the other hand, six isoforms of aquaporins (AQP 1, 3, 4, 5, 8, 9) have been reported in glial cells, performing important functions like water transport, regulation of the cerebrospinal fluid (CSF), synaptic remodeling, formation of brain edemas and inflammatory processes (Saparov et al., 2007; Te Velde et al., 2008; Albertini and Bianchi, 2010; Nagahara et al., 2010). Aquaporin 9 (AQP9) is highly expressed in the inner mitochondrial membrane of astrocytes (Potokar et al., 2016). Different studies have shown that AQP9 is permeable to many solutes including glycerol, purines, pyrimidines and urea, suggesting its importance for diffusion and energetic metabolism in astrocytes (Albertini and Bianchi, 2010; Badaut et al., 2012; Potokar et al., 2016). Moreover, silencing of AQP9 in murine astrocytes decreased glycerol uptake and increased glucose and oxidative metabolism, suggesting its importance for astrocyte metabolism (Badaut et al., 2012). Finally, a study by Nagahara et al. (2010) showed that in synovial tissues from osteoarthritic patients (OA), TNFα regulated AQP9 mRNA and protein expression, thus suggesting that AQP9 could be a biomarker for inflammatory processes (Nagahara et al., 2010). It is possible that a similar mechanism could be present in astrocytes under inflammation; however, additional experimental studies are needed in order to address this issue.

**TABLE 3:** Set of reactions with pro-inflammatory potential, identified through a sensibility analysis for the inflammatory scenario.

**ID**	**Reaction description**	**H. Flux**	**I. Flux**	**Fold change**
FTCD	Formimidoyltransferase cyclodeaminase	0.39	1.28	2.28
H2Otm	H2O transport mitochondrial	−0.26	2.44	10.44

*Fluxes for healthy scenario (H. Flux) and inflammatory scenario (I. Flux) are compared.*

Finally, 8 anti-inflammatory reactions were found to have a change equal or greater that 2-fold when knocked-out in the model (Table 4). 6 of these reactions (r0639, r0653, r0714, r0716, r0718, and r0720) are involved in fatty acid elongation in mitochondria through their association with acyl-CoA (Saparov et al., 2007). This elongation system is responsible for the addition of two carbon units to the carboxyl end of a fatty acid chain, and plays an important role in the maintenance of membrane lipid composition, and in the generation of cell signaling precursors (such as eicosanoids and sphingosine-1 phosphate), energy production, and other unknown pathways related with cancer growth (Murphy et al., 1988; Te Velde et al., 2008).

**TABLE 4:** Set of reactions with anti-inflammatory potential, identified through a sensibility analysis on the inflammatory scenario with palmitate.

**ID**	**Reaction description**	**H. Flux**	**I. Flux**	**Fold change**
AKGMALtm	*a*-ketoglutarate/malate transporter	−0.17	−1.3	−6.85
NADH2_u10 m	NADH dehydrogenase mitochondrial	0.12	0.37	2.17
r0639	Lauroyl-CoA: acetyl-CoA C-acyltransferase	0.02	0.09	4.04
r0653	cMyristoyl-CoA: acetyl-CoA C-myristoyl transferase	0.02	0.09	4.04
r0714	(S)-3-Hydroxyhexadecanoyl-CoA: NAD^+^ oxidoreductase	0.02	0.09	4.04
r0716	(S)-3-Hydroxyhexadecanoyl-CoA hydrolyase	0.02	0.09	4.04
r0718	(S)-3-Hydroxytetradecanoyl-CoA: NAD+ oxidoreductase	0.02	0.09	4.04
r0720	(S)-3-Hydroxytetradecanoyl-CoA hydrolyase	0.02	0.09	4.04

*Fluxes for healthy Scenario (H. Flux) and inflammatory scenario (I. Flux) are compared.*

Our data also showed that AKGMALtm (α-ketoglutarate/malate transporter) experienced a fold change of −6.85 when knocked out (Table 4). This transporter is important for the glutamate/glutamine cycle in astrocytes, which prevents the excessive accumulation of glutamate in the extracellular space and the subsequent excitotoxicity (Hertz, 2013). Finally, the mitochondrial NADH lactate dehydrogenase (LDH) allows lactate use in ATP production in astrocytes during oxidative phosphorylation (Lemire et al., 2008). Recently, it was shown that LDH in murine lymphocyte T cells is important for the T-cell effector functions by increasing histone acetylation and the pro-inflammatory IFN-γ transcription, thus suggesting that LDH could be a therapeutic target in autoinflammatory diseases (Peng et al., 2016). Further *in vivo* and *in vitro* experiments are needed in order to assess this mechanism in astrocytes.

### Tibolone Treatment Scenario

**FIGURE 7:**
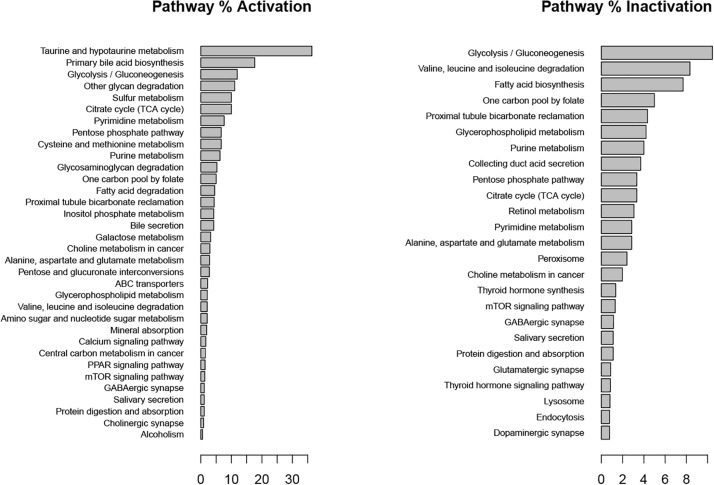
Metabolic pathways affected by tibolone over an inflammatory scenario. Activation and inactivation percentage was measured in comparison with genes associated to each pathway in the KEGG database.

In our “Tibolone treatment” scenario, tibolone affected the flux rate of 948 reactions in comparison with the inflammatory scenario. We found important metabolic changes associated with the activation of several protective pathways in astrocytes (Schuller-Levis and Park, 2003). These include taurine metabolism, which has been shown to protect against oxidative injury in different *in vitro* and *in vivo* models including lung cells, leucocytes, rat macrophages and neuronal cells (Schuller-Levis and Park, 2003), gluconeogenesis which facilitates the conversion of fatty acids into ketone bodies under steroid-mediated effects (Amen-Ra, 2006), calcium, and PPAR signaling path-ways (Figure 7). Interestingly, PPAR gamma has been shown to antagonize the actions of pro-inflammatory transcription factors nuclear factor-κB (NF-κB) and activator protein 1 (AP-1) in human aortic smooth muscle cells and in primary human hepatocytes (Delerive et al., 2000; Daynes and Jones, 2002). These results suggest that tibolone exerts a significative modulation on inflammatory reactions through the activation of several protective pathways, which is agreement with previous experimental results from our group (Avila-Rodriguez et al., 2014, 2016; González-Giraldo et al., 2019).

The “tibolone treatment” scenario also increased the demand for L-aspartate and in turn, decreased the demand for L-asparagine, L-glutamate and the release of L-glutamine when compared with the inflammatory scenario (Figure 3). The reduction on the L-glutamate and L-glutamine uptake/release rate mediated by tibolone could be associated with a neuroprotective effect through the reduction in neurotoxicity mediated by L-glutamate in astrocytes (Petrelli and Bezzi, 2016). In this aspect, it has been shown that the excess in L-glutamate is a contributing factor in neuronal damage induced by inflammation in pathologies like TBI stroke, PD and AD (Ahlemeyer et al., 2002). However, it is important to perform additional simulations in our model to assess supplementary metabolic mechanisms that are associated to the induced inflammation in astrocytes (Shi et al., 2009). Against our initial hypothesis, tibolone treatment did not show actions over reactions affected by inflammation and associated with neuronal support (Table 1). However, tibolone increased cellular growth by 13.26% compared with basal conditions (Figure 6), suggesting an increase either on cell viability or in astrocytic proliferation (Feist and Palsson, 2010). Interestingly, this proliferative potential was not observed in the inflammatory scenario, suggesting that the observed proliferation in our model could be important for cellular homeostasis (Heimann et al., 2017). In this aspect, previous studies have shown that estrogen stimulates the differentiation and proliferation of neural stem cells into neurons, astrocytes, and oligodendrocytes (Okada et al., 2010). There is no evidence of increased proliferation by tibolone under palmitate insult, probably due to the experimental and technical challenges associated with an accurate measurement of cell proliferation (Frago et al., 2017; González-Giraldo et al., 2019).

Based on a sensibility analysis performed over 289 reactions associated with tibolone and estradiol-derived compounds, we identified a set of four reactions that after being individually knocked out, completely blocked tibolone effects in our model (Table 5). The identified reactions are catalyzed by an alcohol dehydrogenase (E.C. 1.1.1.1) and cholestanetriol 26-monooxygenase associated with cytochrome P450 and the PPAR signaling pathway (Mast et al., 2017). Both enzymes were previously reported to be associated with a reduction in ROS production through redox reactions mediated by alcohol dehydrogenase (ADH) and cytochrome P450 (Colditz et al., 1995; Pessayre et al., 2001). Moreover, a recent study in knockout mice showed that cholestanetriol 26-monooxygenase deficiency is associated with early atherosclerosis, osteoporosis, and progressive neurological deterioration associated with AD (Mast et al., 2017). Altogether, these results suggest the importance of tibolone in the regulation of multiple protective mechanisms in the brain.

**TABLE 5:** Set of reactions associated with tibolone, involved in the protective effects of the drug.

**ID**	**Reaction description**	**Genes in astrocyte data**
r0739	Alcohol Dehydrogenase	ADH4, ADH5, ADH7
r2518	ATP-binding Cassette (ABC)	ABCD3
RE1804M	Cholestanetriol 26-monooxygenase	CYP27A1
RE1807M	Cholestanetriol 26-monooxygenase	CYP27A1

*Reactions were identified through a sensibility analysis, carried out in the “Tibolone treatment” scenario.*

## Conclusion

In this work, we developed a tissue-specific metabolic network for human astrocytes that was simulated under three different scenarios. The model allowed us to identify the metabolic changes between a healthy, an inflammatory and a tibolone treatment scenario. In our model, the adverse effects associated with the increase of palmitic acid uptake in astrocytes were described based on exchange fluxes, metabolite production, and metabolic pathways perturbed under the inflammatory response. Moreover, this model was consistent with previous experimental studies showing that tibolone exerts multiple protective effects against inflammation, oxidative stress and metabolic dysregulation (Liu et al., 2013; Avila-Rodriguez et al., 2014, 2016; González-Giraldo et al., 2019. In this aspect, a “Tibolone treatment” scenario was modeled, based on previous works describing the neuroprotection induced by this synthetic compound on astrocytes under a variety of anti-inflammatory stimuli.

Our results suggest that tibolone exerts its protective effects through multiple mechanisms, including the reduction of neurotoxicity mediated by L-glutamate in astrocytes, the activation of inflammatory modulators like PPAR gamma, and the increase in the metabolism of antioxidative molecules like taurine. We also found a tibolone-associated increase in biomass growth rate, which is similar to previously reported studies of the tumorigenic effects exerted by this compound in breast cancer (Colditz et al., 1995). On the other hand, the identified enzymes and reactions associated with tibolone mechanisms are highly consistent with previous results from our lab (Avila-Rodriguez et al., 2014, 2016).

Finally, a sensitivity analysis performed through constrained-based modeling approaches and FBA allowed us to recognize two possible reactions with their associated enzymes, susceptible to be knocked out in order to reduce the inflammatory perturbations of palmitic acid in astrocytes.

In summary, constraint-based models are valuable tools for the study of the protective effects mediated by pharmacological molecules in astrocytes, and provide a detailed insight into high-throughput data analysis. Further experiments are needed in to confirm the involvement of tibolone in the regulation of inflammatory mediators in astrocytic animal and cellular models.

## Data Availability Statement

The datasets generated for this study are available on request to the corresponding author.

## Author Contributions

DO and AP developed the theory, performed the model, contributed to the design and implementation of the research, the analysis of the results, and writing of the manuscript. CM-J and DO performed the simulations and wrote the manuscript with input from all authors. DO, GB, AP, and JG designed the model and the computational framework and analyzed the data. All authors discussed the results and contributed to the final manuscript.

## Conflict of Interest

The authors declare that the research was conducted in the absence of any commercial or financial relationships that could be construed as a potential conflict of interest.

## Footnotes

1 https://vmh.uni.lu

2 https://sourceforge.net/projects/winglpk/files/winglpk/GLPK-4.60/

3 http://himedialabs.com/TD/AT068.pdf
