# Supplementary material for: Multiple Pathways Involved in Palmitic Acid-Induced Toxicity: A System Biology Approach
Source: Front Neurosci. 2020 Jan 31;13:1410. doi: 10.3389/fnins.2019.01410 (PMC7006434; doi:10.3389/fnins.2019.01410)
Supplement: Supplementary file 1 [file Data_Sheet_1.PDF]

Model Capabilities Evaluation

we evaluated metabolic capabilities of the reconstructed network were calculated through various astrocytic metabolic tasks. This metabolic task has important physiological implications. We tested the flux through the different pathways of the metabolic astrocytic network and captured metabolic scenarios developed in astrocytes in different scenarios. The measurement of flux change for each reaction between metabolic scenarios was calculated with the fluxDifferencesfunction

Metabolic task	Reasoning	Changes in Metabolic Fluxes	Bibliographic support
Glutamate uptake	Astrocyte has a main role in glutamate detoxification of the extracellular space	Glutamate-glutamine cycle	(Verkhratsky, Nedergaard, & Hertz, 2014)
Glutamine Release	Export of glutamine from astrocytes, and the uptake of glutamine by neurons, are essential steps in neurotransmission.	Glutamate-glutamine cycle	(Griffin et al., 2003)
Maximization ATP Production	astrocyte metabolism its highly dependent on ATP derived from energetic pathways.	Glycolysis, TCA, oxidative phosphorylation, PPP, amino acids, glutamate, glycine, cysteine and glutamine, glycine, serine-D, reduced glutathione, lactate, and ATP	(Aschner, 2000; Bélanger, Allaman, & Magistretti, 2011; Occhipinti, Somersalo, & Calvetti, 2009)
Minimization ATP Production	To assess the main energetic pathways and the alternative sources of energy that the model would use.	Glycolysis	(Aschner, 2000; Bélanger et al., 2011; Occhipinti et al., 2009)
Blocked Reactions	we set as objective function each one of the reactions (one by time) in the model and identifies the reactions without flux under all scenarios.	Whole model	(Gelius-Dietrich, Desouki, Fritzemeier, & Lercher, 2013)
Energetic pathways	To assess flux and energy production through the all energetic pathways	Glycolysis, TCA, oxidative phosphorylation.	(Kreft, Bak, Waagepetersen, & Schousboe, 2012)
Glycogenesis	Glycogen is found principally in astrocytes.	Glycogenesis- glucose uptake	(Gruetter; Hertz, Peng, & Dienel, 2007)

Maximization glucose uptake	Glycolysis is the main energetic substrate. Astrocytes have a glucose-based metabolism.	Anaerobic glycolysis, TCA, oxidative phosphorylation. Astrocytes activated 52 % of model reactions	(Zwingmann & Leibfritz)
Glucose inhibition	To assess the model behavior with energic depletion	TCA, oxidative and phosphorylation inactive.	(Zwingmann & Leibfritz)
Oxygen inhibition	To assess the model behavior with energic depletion	TCA, oxidative and phosphorylation inactive.	(Zwingmann & Leibfritz)
NADPH	NADPH provides the reducing equivalents.	NADPH producing reaction	(Abramov et al., 2005)
